# Cost‐Effectiveness and Budget Impact Analysis of Apixaban and Rivaroxaban Versus Warfarin in the Prevention of Stroke in Patients With Non‐Valvular Atrial Fibrillation (NVAF) in Iran

**DOI:** 10.1002/clc.24311

**Published:** 2024-06-24

**Authors:** Amirmohammad Tajik, Azam Abbasi, Zahra Goudarzi, Azadeh Izadi‐Moud, Mehdi Varmaghani

**Affiliations:** ^1^ School of Pharmacy Mashhad University of Medical Sciences Mashhad Iran; ^2^ Health Policy Research Center, Institute of Health Shiraz University of Medical Sciences Shiraz Iran; ^3^ Department of Management Sciences and Health Economics, School of Health Mashhad University of Medical Sciences Mashhad Iran; ^4^ School of Health Management and Information Sciences, Health Human Resources Research Center Shiraz University of Medical Sciences Shiraz Iran; ^5^ Department of Cardiovascular Diseases, Faculty of Medicine Mashhad University of Medical Sciences Mashhad Iran; ^6^ Social Determinants of Health Research Center Mashhad University of Medical Sciences Mashhad Iran

**Keywords:** Apixaban, cost‐effectiveness, Iran, Rivaroxaban, stroke, Warfarin

## Abstract

**Introduction:**

This study evaluates the cost‐effectiveness of Apixaban and Rivaroxaban, compared to Warfarin, for stroke prevention in patients with non‐valvular atrial fibrillation in Iran.

**Method:**

A Markov model with a 30‐year time horizon was employed to simulate and assess different treatment strategies' cost‐effectiveness. The study population comprised Iranian adults with NVAF, identified through specialist consultations, hospital visits, and archival record reviews. Direct medical costs, direct nonmedical, and indirect costs were included. Quality‐adjusted life years (QALY) were assessed using an EQ‐5D questionnaire. This study utilized a cost‐effectiveness threshold of $11 134 per QALY.

**Results:**

Apixaban demonstrated superior cost‐effectiveness compared to Rivaroxaban and Warfarin. Over 30 years, total costs were lower in the Apixaban and Rivaroxaban groups compared to the Warfarin group ($126.18 and $109.99 vs. $150.49). However, Apixaban showed higher total QALYs gained compared to others (0.134 vs. 0.133 and 0.116). The incremental cost‐effectiveness ratio for comparing Apixaban to Warfarin was calculated at −1332.83 cost per QALY, below the threshold of $11 134, indicating Apixaban's cost‐effectiveness. Sensitivity analyses confirmed the robustness of the findings, with ICER consistently remaining below the threshold. Over 5 years (2024−2028) of Apixaban usage, the incremental cost starts at USD 70 250 296 in the first year and gradually rises to USD 71 770 662 in the fifth year. DSA and PSA were assessed to prove the robustness of the results.

**Conclusion:**

This study shows that Apixaban is a cost‐effective option for stroke prevention in non‐valvular atrial fibrillation patients in Iran compared to Warfarin.

## Graphical abstract

Apixaban is cost‐effective and significantly reduces thromboembolic events compared to Warfarin and other direct oral anticoagulants in patients with non‐valvular atrial fibrillation.

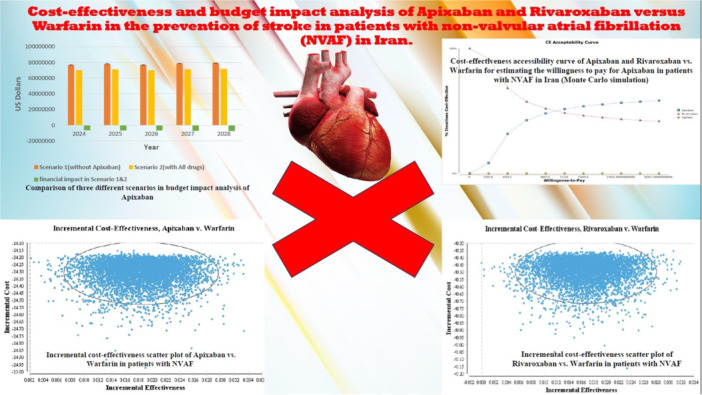

## 1 Introduction

Atrial fibrillation (AF) is the most common type of cardiac arrhythmia in which electrical stimulation does not follow a specific path in the heart [1]. AF occurs when the electrical wave of stimulation in the atria does not have a particular direction; that is, the muscle cells of the atrium are irregularly stimulated and thus contract [2]. As a result, the atria cannot fully pump blood to the ventricles because there is no regular atrium contraction [3]. The ventricular beats do not follow the atrium, and the ventricle contracts without regular order or following and waiting for the atrium [4]. Cardiac arrhythmia is an abnormal heart rhythm that starts at the sinus node and spreads to the ventricles via the atrioventricular node [5]. As a result of this method of conducting electrical stimulation, first, in the atrium and within a short distance, the ventricles contract, and the muscles of the atria relax; then, the ventricles relax [6]. Patients are mostly older adults who also have other diseases [7]. Usually, AF is not life‐threatening but causes other problems [8]. These problems include chronic fatigue, congestive heart failure, and stroke [8].

The incidence and prevalence of AF worldwide is increasing [9]. Statistics show that in the last 20 years, hospitalizations due to rhythm disorders caused by this disease have increased by 66% [10]. This increase is due to the rise in the average age of the world's population, the increase in the prevalence of chronic heart diseases, and the improvement of diagnostic power due to the advancement of equipment [11]. The frequency of the disease varies in different countries, so in the countries of the Western economy, approximately 11 million people are diagnosed with AF [12]. Its prevalence outside the United States, Canada, and Europe varies widely based on age, so it is higher in elderly white populations and much lower in African and South Asian people [13]. AF currently affects about 2.3 million people in the United States and is associated with a fivefold increase in stroke compared to patients without AF [14]. Based on a search conducted in Iran, a study on the prevalence of non‐valvular atrial fibrillation (NVAF) has not been investigated, and its frequency is unknown. However, in some studies, the prevalence of AF was assessed at approximately 0.19% [15].

Cardioembolic stroke includes 15%−20% of the causes of ischemic stroke, and among embolic reasons, the most common underlying cause is AF, which provides for more than half of the cases [16]. Therefore, preventing clots and emboli formation is very important in this disease [17]. The most important and unfortunate complication of AF is the formation of an intra‐atrial clot and embolism (stroke) [18]. The risk of thromboembolism increases up to 20 times in patients with NVAF and seven times in patients with valve diseases and AF [19]. There are about 500 000 strokes related to AF annually worldwide [20].

Globally, more than half of patients with AF require treatment with an oral anticoagulant medicine [21]. Choosing the appropriate medicine treatment requires careful evaluation of the effect of reducing the risk of stroke anticoagulant medication and the possible bleeding effect of that medicine [22].

Warfarin, as an anti‐vitamin K agent (VKA), is the standard oral treatment for stroke prevention in people with NVAF [23]. However, it has many limitations, such as medicine‐food interactions, slow onset of effect, high rates of medicine discontinuation, limited therapeutic range, and variable dose−response of the patient to the medicine, which requires continuous laboratory monitoring [24].

In recent years, several new oral anticoagulants (NOACs), including edoxaban, Rivaroxaban, Apixaban, and dabigatran, have been approved in the United States as an alternative to Warfarin for stroke prevention in NVAF patients [25]. These newer agents provide more convenient and practical anticoagulation therapy because they do not require daily monitoring, have a faster onset of action, and have fewer medicine‐food interactions than VKA therapy [26].

Considering the limited resources of the health sector and since one of the country's most expensive parts of the health system is the pharmaceutical sector, controlling medicine costs is one of the goals of policymakers. One of the requirements to achieve this goal is to conduct economic evaluation studies between two alternative medicines [27, 28, 29, 30]. Therefore, considering that in our country, no analysis has been shown in evaluating the cost‐effectiveness of Apixaban and Rivaroxaban compared to Warfarin, a commonly used anticoagulant, with the Budget impact analysis (BIA), this study aimed to assess the cost‐effectiveness of Apixaban and Rivaroxaban compared to Warfarin to prevent stroke in patients with NVAF.

## 2 Methods

In this study, the Markov model was used to consider the chronic nature of AF and the existence of different disease states, which have the condition of recurrence and return to the initial state in different time sequences. The model drawn in the software is cost‐utility, and costs are estimated in dollars and effectiveness in quality‐adjusted life years (QALY) based on the probabilities entered in the model. This study used Microsoft Excel 2021 and TreeAge 2011 software to analyze the data in different stages. The data was first entered into Excel 2021 software and refined, and then TreeAge 2011 software was used for descriptive and analytical reporting. Therefore, based on the results of the previous stages, the model was entered into the Tree‐age design software and the extracted data, and according to the time horizon of the study, the amount of cost, effectiveness, and cost‐effectiveness was calculated with monetary units, QALY, and cost per QALY, respectively. The ICER of three medicines was calculated and compared.

### 2.1 Model Structure

**Figure 1:**
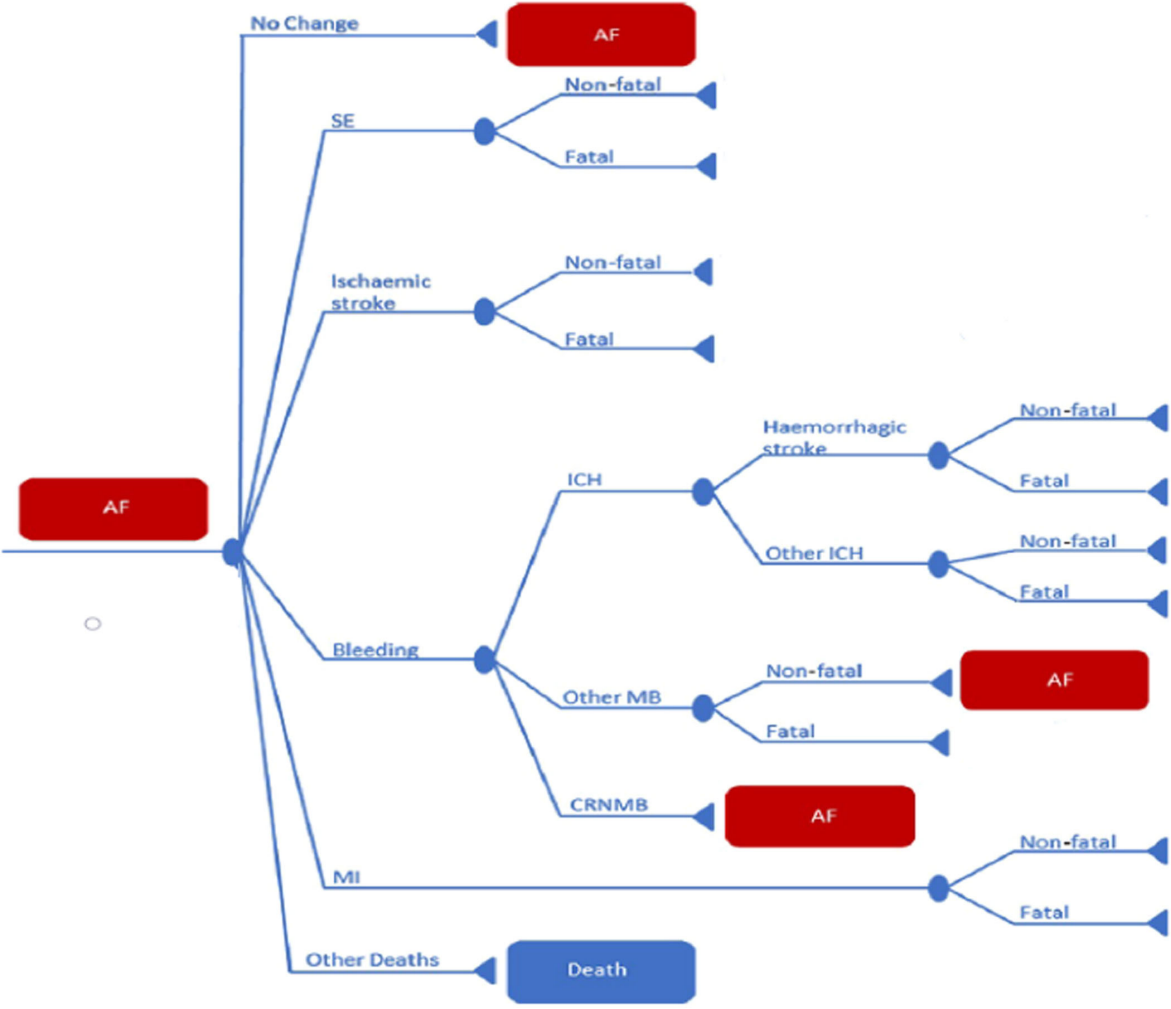
Health state transition diagram for Markov model in atrial fibrillation patients.

An individual‐level simulation model was built to predict each patient's clinical events and outcomes under different treatment regimens over time. The validation and updating of the model were conducted utilizing recent literature data to assess the long‐term financial implications and medical outcomes associated with utilizing apixaban versus vitamin K agent (VKAs) and alternative non‐vitamin K agent oral anticoagulants (NOACs) in Dutch patients with AF [25, 31]. Given the model's structure with apixaban as the baseline treatment, direct comparisons between other NOACs (namely dabigatran, edoxaban, and rivaroxaban) and comparisons between NOACs and VKAs were not feasible [25, 31]. Various health states were incorporated into the model to simulate the progression of AF‐related health conditions over time. Individuals could transition between these health states throughout a hypothetical cohort's lifetime comprising 1000 AF patients. The likelihood of transitioning to another health state, denoted as the transition probability, was computed every 6 weeks and chosen to encompass all relevant AF‐related events within a brief timeframe [25, 31]. Illustrated in Figure 1, the model encompassed the subsequent health states: “AF,” “ischemic stroke,” “systemic embolism” (SE), “bleeding,” myocardial infarction (MI), “other deaths,” and “event‐unrelated anticoagulant discontinuation” (discontinuation of anticoagulation not linked to the specified events). Bleeding episodes were categorized into intracranial hemorrhage (referred to as “ICH,” assumed to comprise “hemorrhagic stroke” and “other ICH”), another major bleeding (“other MB,” encompassing all non‐ICH major bleedings), and clinically relevant nonmajor bleeding (“CRNMB”). Upon experiencing a clinical event, patients remained in the corresponding health state for one cycle before transitioning to either transient or absorbing states. “AF” and “AF without anticoagulant therapy” represented transient states, allowing patients to persist or transition to others within the model, and absorbing states such as “death” indicated terminal states, where patients remained until death.

The study was conducted from the perspective of society, and therefore, direct medical, nonmedical, and indirect costs (IC) were included. Also, the time horizon of the study is based on life expectancy with 1‐month cycles. The year of entering the study was 50 years.

The choice of the 1‐month process was consistent with other published economic evaluation studies [32] and the opinions of clinical experts due to the nature of changes in disease status. Considering that the disease is chronic and the time horizon of the study is more than 1 year, the costs have been discounted at an annual rate of 7.2% and clinical outcomes at a rate of 3%. [33, 34].

### 2.2 Model Inputs

According to Mashhad University of Medical Sciences' health information system (HIS) data, all patients referred to Mashhad Medical Sciences Hospitals with AF problems in 2023 were identified (Supporting Information S1: Table 1). Based on their distribution in different states, the initial distribution of patients in the study was determined. So, the target population for this study consisted of Iranian adults with NVAF.

The first step before collecting data was to identify the patients. The complicated and time‐consuming process of determining the study patients was carried out simultaneously in three ways. Then, according to the entry criteria, the study included the patients. Finally, the required data were collected by two retrospective methods, and a 6‐month follow‐up of the patients was conducted.

Visiting the office of a specialist doctor and plan consultant: The patients identified by this method were of two categories: A group of patients previously diagnosed with AF, and a medical file was prepared for each. A doctor analyzed the second group of patients since the beginning of the plan, and then anticoagulants were prescribed.

Visiting the clinical departments of government hospitals in person: AF patients are admitted to different departments according to the state of their disease when they see the hospital. These departments include acute cardiac departments, cardiac CCU, and internal sensitive; the researcher identified these patients by visiting these departments in person every few days and with the opinion of the relevant doctors and the help of nurses. For example, a patient with AF who suffered from gastrointestinal bleeding following the use of anticoagulants was identified in the endoscopy department and admitted to the internal acute department.

Studying hospital files in the archives and medical records department of government hospitals: in this method, after the researcher provides explanations about the plan to the medical records expert, according to the diagnostic codes registered in the HIS of the hospital, the relevant (and possibly related) files received and then according to the file number registered in the file, the files were taken, and after reading the files, the patients who met the study entry criteria were identified.

It should be noted that hospital records were used because it was possible that during 1 year of follow‐up for newly diagnosed and followed‐up patients, not all disease states would occur, and the obtained data would be incomplete. Therefore, retrospectively, it was decided to include hospital files and AF patients hospitalized due to the study's medicine side effects.

### 2.3 Cost and Resource Data of Quality of Life

To identify the cost items, after consulting with the relevant experts in the disease and its treatment protocol and considering the perspective of the study, a cost checklist was prepared in the form of a questionnaire and was approved by the consultants. The questionnaire was completed through patients' self‐reports (phone calls or face‐to‐face interviews with patients) and medical and financial documents available in hospitals (for more accuracy regarding hospitalization costs). A bottom‐up approach has been used to calculate costs. Overall, the study considered all direct, nonmedical, and indirect medical costs. (Supporting Information S1: Table 2). Direct medical costs (DMC) included the cost of the medicine, the cost of a physician, the cost of diagnostic testing, the cost of diagnostic imaging, and other medical costs. Direct nonmedical costs (DNMC) consist of expenditures associated with transportation, both local and long‐distance, as well as food and lodging for the patient and their accompanying individuals. This category also covers the acquisition of medical equipment and devices (like wheelchairs, walkers, and home care beds) and adjustments to the patient's home necessitated by the illness. The IC cover lost productivity resulting from workforce absence, which includes early retirement and premature death. The human capital approach was employed to assess these IC (lost income), where the financial worth of reduced productivity due to disability or death was based on the individual's pre‐disability and pre‐death salary. The calculation for IC only considered income loss and mortality expenses for individuals under the age of 65 and subjects, and the cost of wages and salaries for healthcare providers caring for patients. Multiply the minimum daily wage by the total number of days of disability for patients and their caregivers to determine the IC. The average daily wage for laborers in Iran is 1 769 428 Iranian Rials (equivalent to USD 1 = IRR 409 841). The minimum monthly salary is 53 082 840 Rials (equivalent to USD 129.52), while the minimum daily wage is equivalent to USD 4.32 [35, 36].

All costs were calculated in 2023 US Dollars, with an exchange rate of USD 1 = IRR 409 841 (Iranian Rial). The study utilized a cost‐effectiveness threshold of 11 134 USD, as the WHO recommended, based on the latest acceptable CEA threshold announced by the Iran Food and Drug Association.

Quality of life scores were obtained using the standard EQ‐5D questionnaire through face‐to‐face or telephone interviews with patients, and then health outcomes were determined in the form of QALY. Therefore, finally, the number of years of QALY's life expectancy was calculated using the product of the average utility obtained in each disease state.

### 2.4 Transition Probabilities

The data relating to the probabilities, which include clinical events and are usually beyond the doctor's control, are extracted from the review of related papers [37]. Due to the limitation of data, especially clinical data, according to the medicine treatment protocols used in AF patients in the country, the transfer probabilities between health states were extracted from studies and international evidence in NVAF [31]. Table 1 shows the transition probabilities used.

**Table 1:** Rates of events for Apixaban, Rivaroxaban, and VKA, along with the breakdown of patients across various levels of severity for both ischemic and hemorrhagic strokes.

State	Event rate per 100 PY			References
	Apixaban	VKA	Rivaroxaban	
Ischemic stroke				
CHADS2	0.950 (0.719−1.212)	0.950 (0.791−1.250)	1.030 (0.770−1.380)	[38, 39, 40, 41, 42, 43, 44, 45, 46, 47]
ICH				
CHADS2	0.348 (0.199−0.538)	0.582 (0.612−1.176)	1.730 (1.080−2.770)	[38, 39, 40, 41, 42, 43, 44, 45, 46, 47]
Other MB				
CHADS2	1.898 (1.085−2.935)	2.360 (1.349−3.446)	1.410 (1.130−1.750)	[38, 39, 40, 41, 42, 43, 44, 45, 46, 47]
CRNMB	2.083 (1.191−3.221)	2.995 (2.625−3.562)	1.520 (1.280−10)	[38, 39, 40, 41, 42, 43, 44, 45, 46, 47]
MI	0.530 (0.303−0.820)	0.610 (0.461−0.811)	1.060 (0.730−1.520)	[38, 39, 40, 41, 42, 43, 44, 45, 46, 47]
SE	0.090 (0.051−0.139)	0.100 (0.057−0.155)	0.850 (0.350−2.060)	[38, 39, 40, 41, 42, 43, 44, 45, 46, 47]
Other CV hospitalization	10.460 (5.979−16.174)	10.460 (5.979−16.174)	1.000 (0.900−1.100)	[48]

Abbreviations: AC, anticoagulant; CHADS_2_, congestive heart failure, hypertension, age ≥75 years; CRNMB, clinically relevant nonmajor bleeding; CV, cardiovascular; ICH, intracranial hemorrhage; MB, major bleed; MI, myocardial infarction; PY, patient‐years; SE, systemic embolism; VKA, vitamin K agent.

### 2.5 Baseline Analysis

The research utilized the incremental cost‐effectiveness ratio (ICER) index to assess and identify the strategy that offers the best value for money. To determine cost‐effectiveness, the assessment followed the Ministry of Health's guidelines and assumed that a threshold equal to Iran's per capita Gross Domestic Product in 2023, amounting to 700 million Rial (USD 11 134 based on the purchasing power parity [PPP] index), should be met. To account for varying currency values, the cost variables in this study were converted into Rial using the PPP conversion factor, which is equivalent to USD 62 870.

### 2.6 Sensitivity Analysis

In this research, deterministic sensitivity analysis (DSA) and probabilistic sensitivity analysis (PSA) were employed to examine the impact of parameter uncertainty on the model outcomes. DSA involved ±20.0% adjustments in essential data points of costs and 10.0% variation in the probabilities and utilities of these patients in the first year were considered, while PSA utilized Monte Carlo simulation with 5000 iterations to produce a scatter plot and acceptability curve, addressing the uncertainties within the model.

### 2.7 BIA

A Markov‐based model was utilized to assess the budget impact of introducing Apixaban as a treatment option for individuals with NVAF within the Iranian healthcare framework. The analysis included a comparison of Apixaban with Rivaroxaban and Warfarin. The study took into account the population of NVAF patients and their prevalence among individuals aged 50 and above in Iran. Market share estimations were based on official pharmaceutical data provided by the Food and Medicine Organization of the Ministry of Health in Iran. Three scenarios were developed to evaluate the budget impact of incorporating Apixaban. The first scenario was Market share in the absence of Apixaban, continuing treatment with alternative medications. The second scenario was the Inclusion of all available medications in the market, and the final scenario was Market share without Rivaroxaban, with treatment continuing using other available medicines.

## 3 Result

### 3.1 Base Case Analysis

According to the results, over a 30‐year period, the total costs were higher in the Warfarin group compared to the Apixaban and Rivaroxaban groups ($150.49 vs. $126.18 and $109.99). However, the Apixaban and Rivaroxaban groups experienced higher total QALYs gained compared to Warfarin (0.134 and 0.133 vs. 0.116), resulting in an incremental QALY of 0.01 and 0.18. The ICER for comparing Apixaban and Rivaroxaban to Warfarin was showed that Apixaban and Rivaroxaban are dominant against Warfarin (Table 2).

**Table 2:** Base‐case analysis results (per patient).

Strategy name	Total cost ($)	Total QALYs	ICER	Incr cost ($)	Incr QALYs	Subset	ACER (USD/QALY)
Rivaroxaban	109.99	0.133	0	0	0	Undominated	827.197
Apixaban	126.18	0.134	9947.8	0.0016	16.185	Undominated	937.448
Warfarin	150.49	0.116	−1332.83	−0.0182	24.311	abs. dominated	1293.331

Abbreviations: ACER, average cost‐effectiveness ratio; ICER, incremental cost‐effectiveness ratio; Incr, incremental; QALY, quality‐adjusted life year.

### 3.2 Sensitivity Analysis Results

#### 3.2.1 DSA

The Tornado diagram (Supporting Information S1: Figure 1a,b) presents the outcomes of the DSA of Apixaban and Rivaroxaban versus Warfarin. These findings indicate that the ICER consistently stays below the USD 11 134 per QALY threshold across all analyses.

#### 3.2.2 PSA

**Figure 2:**
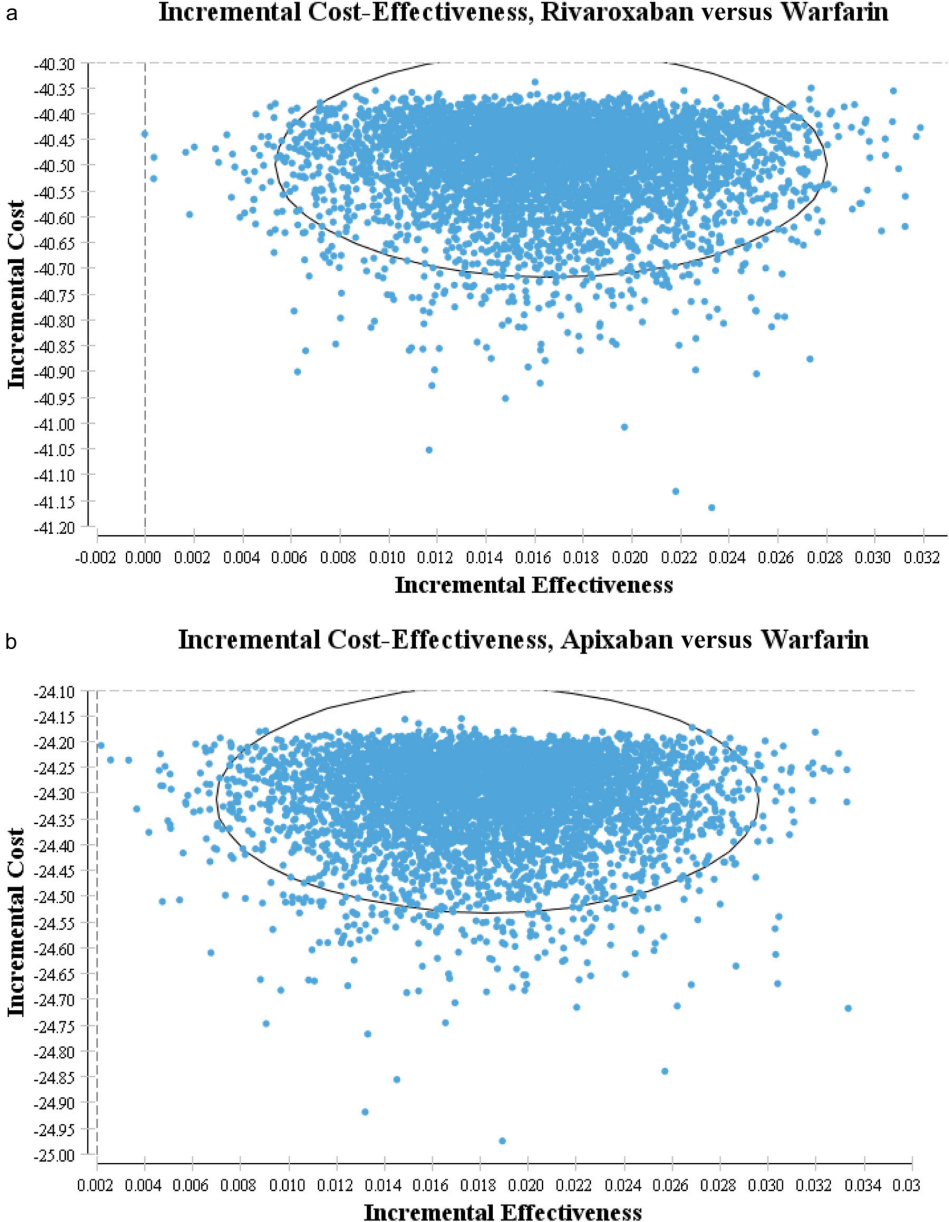
(a) Incremental cost‐effectiveness scatter plot of Apixaban versus Warfarin in patients with NVAF. (b) Incremental cost‐effectiveness scatter plot of Rivaroxaban versus Warfarin in patients with NVAF.

The scatter plot (Figure 2a), known as the Incremental Cost‐Effectiveness plot, compares the strategies of Apixaban and Warfarin. After conducting over 5000 simulation repetitions, the analysis reveals a strong likelihood (over 99%) of Apixaban compared with Warfarin positioned in the optimal areas of the cost‐effectiveness scatter plot, below the threshold line for cost‐effectiveness. Similarly, the probability of Rivaroxaban being considered cost‐effective compared to Warfarin is approximately 100%. Additionally, there is about a 51% chance that it is deemed cost‐effective compared to Apixaban versus Rivaroxaban (Figure 2b). These findings further reinforce the robustness of the base case analysis.

According to Supporting Information S1: Figure 2 of the Cost‐Effectiveness Acceptability curve, as the cost‐effectiveness threshold increases, the chances of cost‐effective Apixaban strategies increase while those of Rivaroxaban decrease. However, both Rivaroxaban and Apixaban strategies remain consistently favorable alternatives to Warfarin at all cost‐effectiveness threshold levels. Overall, the DSA and PSA findings confirm the reliability of the base case analysis results. Supporting Information S1: Table 3 provides a summary of the variables used in the DSA and PSA.

#### 3.2.3 BIA

Table 3 presents the data used to estimate the expenses associated with Apixaban. The total cost of treating patients was calculated by considering the number of patients and the treatment cost per patient. The results indicate that including Apixaban in Iran's medicine list does not impose a significant financial burden on the healthcare system. In the initial year (2024), the projected cost of treating AF without Apixaban was approximately USD 76 663 950. However, incorporating Apixaban and other medicines increased the cost to roughly USD 70 250 296. This upward trend persisted in the following years. It is important to note that while adding Apixaban to Iran's medicine list will result in increased costs for the country and society, it will also improve patient treatment and quality of life. Over 5 years (2024−2028) of Apixaban usage, the incremental cost starts at USD 70 250 296 in the first year and gradually rises to USD 71 770 662 in the fifth year (refer to Supporting Information S1: Figure 3a,b).

**Table 3:** BIA analysis results.

Year	2024	2025	2026	2027	2028
Iran population	86 369 815	87 406 253	88 455 128	89 516 589	90 590 788
Total population	30 471	30 837	31 207	31 581	31 960
Market share of Warfarin	0.21	0.2	0.18	0.18	0.17
Market share of Apixaban	0.29	0.30	0.30	0.31	0.32
Market share of Rivaroxaban	0.38	0.39	0.40	0.40	0.40
Market share of LMWH	0.12	0.12	0.11	0.11	0.11
Scenario 1 (without Apixaban)	76 663 950	78 014 031	76 681 764	78 431 527	79 193 663
Scenario 2 (with all medicines)	70 250 296	71 299 598	69 886 759	71 325 764	71 770 662
Scenario 3 (without Rivaroxaban)	77 779 016	79 119 164	78 003 068	79 539 468	8 0082 930
Financial impact in scenario 1 & 2	−6 413 654	−6 714 432	−6 795 005	−7 105 764	−7 423 002
Financial impact in scenario 1 & 3	−7 528 720	−7 819 566	−8 116 309	−8 213 704	−8 312 269

Abbreviation: LMWH, low molecular weight heparin.

## 4 Discussion

This study employs a Markov model to explore the chronic nature of AF, accounting for various disease states and their recurrence patterns. Utilizing cost‐utility analysis, costs are estimated in dollars, and effectiveness is measured in QALYs, with data analyzed using Excel and TreeAge 2011 software. The Rivaroxaban strategy emerges with the lowest average cost, contrasting with Warfarin's highest average cost, while the Apixaban strategy demonstrates the highest QALY value. Over a 30‐year period, Warfarin incurs higher total costs compared to Apixaban and Rivaroxaban. However, Apixaban yields greater total QALYs gained, making it incrementally cost‐effective compared to Rivaroxaban, with an ICER of 9947.8 QALY/$, which is below the predetermined threshold of $11 134 per QALY. Sensitivity analyses reinforce the robustness of these findings, with both Rivaroxaban and Apixaban strategies consistently emerging as favorable alternatives to Warfarin across various scenarios. Incorporating Apixaban into Iran's medicine list appears financially feasible, with projected costs increasing gradually over 5 years while improving patient outcomes and quality of life.

While numerous investigations have explored the cost‐effectiveness of novel oral anticoagulants (NOACs) for preventing stroke caused by atrial NVAF in high‐income countries [49, 50, 51, 52], our research appears to be among the limited ones addressing this matter in lower and middle‐income countries (LMICs), specifically offering the first comprehensive economic assessment in Iran and the EMRO region (Eastern Mediterranean Region) regarding NVAF patients [53]. The study's objective is to assess the societal perspective of implementing new oral anticoagulant strategies for stroke prevention.

After analyzing various studies conducted in different countries, it is clear that Apixaban and Rivaroxaban demonstrated higher effectiveness than Warfarin, supporting the current study's findings [26, 29, 31, 42, 45, 47, 54, 55]. According to the results of our research that compared the effectiveness of three medications, Rivaroxaban, Apixaban, and Warfarin, it was found that Apixaban emerges as a more cost‐effective option. The findings of this study support the results of other similar studies that have been conducted previously. When we examine the findings from different studies on the cost‐effectiveness of Apixaban or Rivaroxaban compared to Warfarin, varying cost‐effectiveness ratios have been documented. There could be several reasons for this discrepancy. One major factor is the variation in healthcare cost estimation methods across countries with diverse healthcare systems, service delivery structures, and pricing mechanisms. Additionally, using different short‐ or long‐term models and varying cycle lengths to measure costs and outcomes may significantly contribute to the observed differences. For example, the de Jong et al. study employed a 6‐week cycle length, while the Harrington et al. and Lee et al. studies used a 1‐month cycle length in their models [32, 54, 55]. Also, in the UK survey conducted by Kevin Bowrin, a cycle length of 3 months over a lifetime horizon was considered [56]. Therefore, based on the varying cost‐effectiveness values observed in these studies and the present study, it is evident that differences in perspective, cost calculation methodologies, timing of outcome assessments, and patient quality of life assessments ultimately influence the final determination of medicine efficacy and cost‐effectiveness comparisons.

In cost‐effectiveness assessments, the compared treatments are assumed to be perfect substitutes, meaning they would be interchangeable. However, the treatments may be prescribed to slightly different patients in real‐world settings. In Iran [57, 58] and elsewhere [59, 60], direct oral anticoagulants (DOAC)‐treated patients have been younger and have fewer comorbidities than patients treated with warfarin. In Iran, patients using apixaban and warfarin appear to be at increased risk of thromboembolic complications when compared to users of dabigatran and rivaroxaban [59]. In addition, apixaban is more frequently prescribed to women and patients with a prior history of MI [59]. Even factors not related to a patient's disease characteristics may influence treatment choices in real life. Current evidence suggests there may be inequalities in access to DOACs as DOAC‐treated patients tend to have higher education and income levels [60, 61]. In our study‐based analysis, the relative treatment effects were obtained from analyses where apixaban was separately compared to each comparator using propensity score matching [62]. Because of this, the current study should not be interpreted to provide evidence of the relative cost‐effectiveness between the other three anticoagulants. Furthermore, it should be kept in mind that the results may not be generalizable to other countries since country‐specific differences (e.g., drug prices and costs of other healthcare resources) influence the results.

This study involved developing models to evaluate the financial implications of using Apixaban for preventing strokes in patients with NVAF over 5 years. Following guidelines from the International Society for Pharmacoeconomics and Outcomes Research, a time frame was established for conducting the BIA [63]. The findings from this investigation suggest that under scenarios one and two, the budgetary impact is projected to rise annually from 2024 to 2028. Utilizing this analytical method can assist policymakers in middle‐ and low‐income nations in making informed decisions about optimizing resource allocation within their health program planning.

The present study stands out for its notable strengths. First, it comprehensively covers all costs, including DMC, DNMC, and IC. Furthermore, it represents the pioneering research endeavor in Iran to focus exclusively on NVAF patients. Additionally, it utilizes cost and effectiveness data that are native to the context, thereby amplifying the study's validity and relevance. However, there are a few limitations that need to be considered while interpreting the findings of this study. The study participants were difficult to monitor, and their cooperation was limited, which made it challenging to collect and calculate quality‐of‐life data. As a result, quality‐of‐life data was collected only twice—at the beginning and end of the study. It is important to note that these patients' average quality of life in subsequent measurements may vary. However, efforts were made to overcome this issue by increasing the study's sample size. The careful selection of appropriate samples helped reduce concerns related to variations in quality of life. In addition, newer oral anticoagulants have not been compared directly in head‐to‐head studies. Therefore, economic analysis relies on indirect comparisons through network meta‐analysis, which requires minimizing differences in clinical and methodological aspects across studies. Additionally, the efficacy and safety data for Apixaban and other comparable models were sourced from extensive clinical trials conducted beyond Iran, potentially not fully representative of the effectiveness and safety of AF treatments within the Iranian population. Nevertheless, these trials encompassed a diverse range of patients from various backgrounds, leading to the assumption that the efficacy and safety outcomes would align similarly within the Iranian population.

## 5 Conclusion

The results of this research endorse the utilization of Apixaban and Rivaroxaban over Warfarin as a cost‐efficient therapy choices for treating NVAF in Iran. It is recommended that future studies with increased sample sizes and broader patient demographics be carried out to validate these results. Additionally, it is essential to assess the cost‐effectiveness of Rivaroxaban compared to other NOACs for the treatment of valvular and NVAF. Overall, to gain a better understanding of the financial impact of AF, it is highly recommended to gather comprehensive demographic data on patients with this condition, as well as their treatment history. Such information can provide a more nuanced and precise estimation of the costs associated with this disease, including healthcare expenses and lost productivity due to missed work or reduced quality of life. By having a clearer picture of AF patients' demographics and treatment patterns, healthcare providers and policymakers can make more informed decisions about resource allocation, treatment options, and public health initiatives. Access to epidemiological insights and accurate cost projections can greatly assist decision‐makers in prioritizing resource allocation and policy formulation.

## Author Contributions

A.T., M.V., and A.A. contributed to the designing and running of the model. A.T. and A.A. gathered the required data for the model, including literature review, cost, and efficacy data. A.T. and M.V. contributed to the study's statistical and epidemiological parts and double‐checked all analyses. A.T., M.V., and A.A. wrote the manuscript. Z.G. and A.I.‐M. reviewed the manuscript and double‐checked all the analyses. All authors participated in reviewing the manuscript and its revision, and they were involved in research, interpretation, and finalizing the manuscript.

## Ethics Statement

Ethical approval was not required as this study's data were neither confidential nor commercially sensitive. However, the study has been approved by the Ethics Committee of Mashhad University of Medical Sciences with the code of IR.MUMS.FHMPM.REC.1402.237. Relevant guidelines and regulations are carried out for all methods.

## Consent

Informed consent was obtained from all subjects.

## Conflicts of Interest

The authors declare no conflicts of interest.

## Supporting information

Supporting information.

## Data Availability

The data sets used and analyzed during the current study are available from the corresponding author upon reasonable request.
