# Supplementary material for: Cost‐Effectiveness and Budget Impact Analysis of Apixaban and Rivaroxaban Versus Warfarin in the Prevention of Stroke in Patients With Non‐Valvular Atrial Fibrillation (NVAF) in Iran
Source: Clin Cardiol. 2024 Jun 24;47(6):e24311. doi: 10.1002/clc.24311 (PMC11194975; doi:10.1002/clc.24311)
Supplement: Supplementary file 1 — Supporting information. [file CLC-47-e24311-s001.docx]

Supplementary Table 1 Demographic characteristics (based on sex) of subjects participating in the study

| Demographic Information | | |
| --- | --- | --- |
| Sex | Female | 48 (42%) |
|  | Male | 67 (58%) |
| Insurance Tape | Iranian Health Insurance | 70 (61%) |
|  | Social Security Insurance | 42 (36%) |
|  | Armed Forces Insurance | 3 (3%) |
| Supplementary insurance | YES | 4 (3%) |
|  | NO | 111 (97%) |
| age | Minimum | 50 |
|  | maximum | 92 |
|  | average | 68.3 |
|  | Standard Deviation | 14.9 |

Supplementary Table 2 Direct and indirect costs applied in the base-case

| **Warfarin** | **Well With AF** | **MI** | **Stroke** | **GI Bleeding** | **SE** |
| --- | --- | --- | --- | --- | --- |
| **cost of the medicine** | 14.86 (2.38-70.79) | 81.43 (5.36-571.31) | 198.31 (0.88-3870.81) | 33.51 (1.30-2756.64) | 122.95 (42.96-375.29) |
| **cost of physician** | 23.40 (4.81-49.45) | 331.82 (111.45-785.69) | 251.52 (15.84-2190.35) | 59.79 (5.39-581.65) | 63.40 (12.37-143.03) |
| **cost of diagnostic testing** | 14.18 (0.41-23.32) | 21.33 (0.40-107.41) | 67.840.11577.42) | 34.04 (0.02-506.24) | 33.91 (0.88-110.29) |
| **cost of diagnostic imaging** | 13.20 (8.49-21.20) | 26.00 (10.70-57.17) | 52.4922.22154.68) | 36.34 (13.83-122.33) | 20.73 (11.34-42.02) |
| **other medical costs** | 1.53 (0.11-8.23) | 22.11 (11.71-54.32) | 29.082.8260.17) | 25.65 (11.20-99.12) | 3.41 (0.29-10.96) |
| **cost of the inpatient** | 197.73 (16.10-1028.54) | 230.27 (3.34-3261.94) | 525.3717.244236.92) | 643.37 (106.85-6400.70) | 539.47 (7.24-168.15) |
| **cost of the Auxiliary** | 67.17 (16.20-173.00) | 482.68 (139.61-1575.90) | 599.2341.857053.43) | 189.33 (31.74-4065.98) | 244.39 (67.84-681.59) |
| **cost of alterations of property** | 0.00 | 31.37 (23.56-87.05) | 104.5623.56135.76) | 31.37 (20.59-112.8) | 28.23 (23.56-69.12) |
| **transportation cost** | 4.73 (3.29-7.81) | 13.92 (7.81-26.00) | 3112.6967.47) | 13.92 (9.73-25.12) | 13.92 (12.04-30.12) |
| **time lost from work by family members** | 56.12 (24.40-183.00) | 232.67 (118.13-355.63) | 966.17207.493893.58) | 232.67 (118.13-412.69) | 231.64 (118.13-355.63) |
| **cost of medical care** | 9.15 (0.00-36.60) | 100.94 (26.26-179.03) | 621.87361.281292.88) | 107.32 (26.00-182.45) | 117.65 (35.85-193.69) |
| **Utility (EQ-5D-TTO)** | 0.75 (0.64-0.85) | 0.54 (0.39-0.66) | 0.520.330.64) | 0.66 (0.66-0.67) | 0.50 (0.45-0.67) |
| **Rivaroxaban** | **Well With AF** | **MI** | **Stroke** | **GI Bleeding** | **SE** |
| **cost of the medicine** | 3.91 (3.02-4.93) | 37.85 (4.38-120.27) | 52.25 (1.12-160.02) | 8.83 (1.65-113.96) | 32.40 (15.51-54.41) |
| **cost of physician** | 10.83 (9.10-14.35) | 196.94 (118.83-428.99) | 116.37 (62.90-635.61) | 27.66 (21.40-168.79) | 29.34 (49.11-41.5) |
| **cost of diagnostic testing** | 1.39 (1.02-2.69) | 5.24 (0.76-22.51) | 6.65 (0.71-25.36) | 3.34 (0.14-22.24) | 3.33 (5.8-14.84) |
| **cost of diagnostic imaging** | 15.16 (9.82-17.32) | 26.88 (10.76-54.90) | 60.27 (25.70-126.35) | 41.73 (16.00-99.93) | 23.80 (13.12-34.33) |
| **other medical costs** | 1.74 (0.28-3.51) | 56.71 (54.39-64.78) | 33.10 (7.03-110.85) | 29.19 (28.14-42.23) | 3.88 (0.74-4.67) |
| **cost of the inpatient** | 236.63 (18.95-1210.05) | 270.91 (3.93-3837.58) | 618.08 (20.28-4984.61) | 219.48 (77.33-593.93) | 57.36 (50.05-57.9) |
| **cost of the Auxiliary** | 33.03 (24.91-41.13) | 323.61 (189.12-691.45) | 268.65 (97.47-1058.19) | 110.75 (67.33-447.14) | 92.74 (84.29-139.75) |
| **cost of alterations of property** | 0.00 | 30.05 (20.17-72.42) | 102.12 (20.23-104.67) | 29.17 (20.59-112.00) | 27.81 (7.63-53.65) |
| **transportation cost** | 4.49 (3.54-5.12) | 13.92 (15.01-26.00) | 27.55 (12.69-66.83) | 14.64 (9.73-27.59) | 14.48 (7.80-48.5) |
| **time lost from work by family members** | 56.12 (24.40-86.25) | 230.45 (93.79-397.47) | 866.58 (17.80-3482.77) | 230.00 (118.13-397.66) | 233.99 (116.12-324.47) |
| **cost of medical care** | 0.00 | 100.94 (23.82-186.35) | 571.73 (324.49-1253.72) | 112.72 (24.96-171.73) | 115.80 (33.63-182.84) |
| **Utility (EQ-5D-TTO)** | 0.80 (0.66-0.84) | 0.75 (0.60-0.82) | 0.75 (0.60-0.83) | 0.80 (0.75-0.84) | 0.75 (0.60-0.85) |
| **Apixaban** | **Well With AF** | **MI** | **Stroke** | **GI Bleeding** | **SE** |
| **cost of the medicine** | 5.21 (1.16-12.22) | 61.47 (3.19-468.38) | 69.58 (0.43-668.07) | 11.76 (0.63-475.77) | 43.14 (20.95-64.77) |
| **cost of physician** | 9.07 (3.11-19.77) | 216.76 (67.54-575.14) | 97.52 (10.23-875.45) | 23.18 (3.48-232.47) | 24.58 (7.99-57.17) |
| **cost of diagnostic testing** | 1.53 (0.42-3.09) | 9.38 (0.22-67.91) | 7.33 (0.11-76.52) | 3.68 (0.02-67.09 | 3.66 (0.91-14.62) |
| **cost of diagnostic imaging** | 11.5 (8.10-15.04) | 21.9 )10.15-42.24) | 45.72 (21.19-109.76) | 31.66 (13.20-86.81) | 18.06 (10.82-29.82) |
| **other medical costs** | 11.73 (1.83-16.85) | 66.18 (42.13-93.21) | 223.21 (46.79-532.31) | 196.84 (187.24-202.80) | 26.16 (4.91-22.42) |
| **cost of the inpatient** | 255.89 (20.84-1331.05) | 298.00 (4.32-4221.34) | 679.89 (22.31-5483.07) | 281.26 (8.95-1216.09) | 73.49 (49.85-102.48) |
| **cost of the Auxiliary** | 39.05 (14.62-66.96) | 375.69 (123.23-1246.89) | 443.36 (78.76-2262.10) | 267.11 (204.57-1064.94) | 115.60 (45.58-188.79) |
| **cost of alterations of property** | 0.00 | 4.67 (19.83-49.60) | 101.88 (21.77-102.44) | 33.80 (19.76-107.09) | 29.169.450.14) |
| **transportation cost** | 5.71 (4.64-7.93) | 13.00 (9.40-27.72) | 33.39 (10.24-70.01) | 12.25 (7.99-44.96) | 12.267.3847.22) |
| **time lost from work by family members** | 60.76 (24.40-107.85) | 242.84 (96.76-421.14) | 853.55 (19.50-3394.68) | 229.26 (107.10-365.57) | 241.56105.54307.1) |
| **cost of medical care** | 0.00 | 100.00 (23.96-175.55) | 535.25 (309.48-1244.20) | 99.29 (31.38-175.15) | 98.88 (36.24-173.13) |
| **Utility (EQ-5D-TTO)** | 0.8 (0.75-0.84) | 0.75 (0.60-0.82) | 0.750 (0.60-0.83) | 0.80 (0.75-0.84) | 0.75 (0.60-0.85) |

Supplementary Table 3**:** Model Parameter

| **Parameters** | **Base-case value** | **Range** | **Distribution** | **References** |
| --- | --- | --- | --- | --- |
| Age of patient | Under 18 years | Fixed | Fixed |  |
| Cost discount rate per annum | 7.2% | 3.0%-10.0% | Fixed | 49 |
| Effects discount rate per annum | 5.0% | 3.0%-10.0% | Fixed | 50 |
| **DSA** | | | | |
| Cost of the auxiliary in MI in Warfarin | 482.68 | (386.14- 579.22) | Fixed |  |
| Cost of the auxiliary in MI in Apixaban | 375.69 | (300.52- 450.83) | Fixed |  |
| Cost of the auxiliary in MI in Rivaroxaban | 323.61 | (258.89-388.33) | Fixed |  |
| Cost of the auxiliary in SE in Apixaban | 115.60 | (92.48-138.72) | Fixed |  |
| Cost of the auxiliary in SE in Rivaroxaban | 92.74 | (74.19-111.29) | Fixed |  |
| Cost of the auxiliary in SE in Warfarin | 244.39 | (195.51-293.27) | Fixed |  |
| Cost of the auxiliary in stroke in Apixaban | 443.36 | (354.69-532.03) | Fixed |  |
| Cost of the auxiliary in stroke in Rivaroxaban | 268.65 | (214.92-322.38) | Fixed |  |
| Cost of the auxiliary in stroke in Warfarin | 599.23 | (479.38-719.08) | Fixed |  |
| Cost of the medicine in SE in Apixaban | 43.14 | (34.51-51.74) | Fixed |  |
| Cost of the medicine in SE in Rivaroxaban | 32.40 | (25.92-38.88) | Fixed |  |
| Cost of the medicine in SE in Warfarin | 122.95 | (98.36- 147.54) | Fixed |  |
| Cost of the medicine in hemorrhagic stroke in Apixaban | 59.53 | (47.62- 71.43) | Fixed |  |
| Cost of the medicine in hemorrhagic stroke in Rivaroxaban | 44.71 | (35.77- 53.61) | Fixed |  |
| Cost of the medicine in hemorrhagic stroke in Warfarin | 169.67 | (135.74- 203.6) | Fixed |  |
| Cost of the inpatient of ICH stroke in Apixaban | 141.02 | (112.82- 169.22) | Fixed |  |
| Cost of the inpatient of ICH stroke in Rivaroxaban | 110.07 | (88.05- 132.17) | Fixed |  |
| Cost of the inpatient of ICH stroke in Warfarin | 322.64 | (258.11- 387.17) | Fixed |  |
| Cost of the other stroke in Apixaban | 223.21 | (178.57- 267.58) | Fixed |  |
| Cost of the other stroke in Rivaroxaban | 33.10 | (26.48-39.7) | Fixed |  |
| Cost of the other stroke in Warfarin | 29.07 | (23.26-34.88) | Fixed |  |
| Probability of MI_nonfatal in Apixaban | 0.0432 | (0.0388- 0.0475) | Fixed |  |
| Probability of MI_nonfatal in Rivaroxaban | 0.0845 | (0.0768- 0.0939) | Fixed |  |
| Probability of MI_nonfatal in Warfarin | 0.0495 | (0.0445-0.0544) | Fixed |  |
| Probability of bleeding_ICH_hemorrhagic stroke_fatal in Apixaban | 0.0287 | (0.0229-0.0316) | Fixed |  |
| Probability of bleeding_ICH_hemorrhagic stroke_fatal in Rivaroxaban | 0.0222 | (0.0199-0.0244) | Fixed |  |
| Probability of bleeding_ICH_hemorrhagic stroke_fatal in Warfarin | 0.0432 | (0.0388- 0.0475) | Fixed |  |
| **PSA** | | | | |
| **Utilities** | **Mean** | **SD** | **Distribution** | **reference** |
| Utility in MI in Warfarin | 0.54 | 0.068 | Beta |  |
| Utility in MI in Apixaban | 0.75 | 0.07 | Beta |  |
| Utility in MI in Rivaroxaban | 0.75 | 0.07 | Beta |  |
| Utility in stroke in Warfarin | 0.52 | 0.078 | Beta |  |
| Utility in stroke in Apixaban | 0.75 | 0.058 | Beta |  |
| Utility in stroke in Rivaroxaban | 0.75 | 0.058 | Beta |  |
| Utility in SE in Warfarin | 0.50 | 0.04 | Beta |  |
| Utility in SE in Rivaroxaban | 0.75 | 0.055 | Beta |  |
| Utility in SE in Apixaban | 0.75 | 0.055 | Beta |  |
| Cost of the medicine in SE in Warfarin | 122.95 | 83.92 | Gama |  |
| Cost of the medicine in SE in Rivaroxaban | 32.40 | 9.8 | Gama |  |
| Cost of the medicine in SE in the Apixaban | 43.14 | 11.06 | Gama |  |
| **transitional probabilities** | | | | |
| Initial distribution of_AF_Apixaban | 0.0513 |  | Fixed |  |
| Initial distribution of SE in Apixaban | 0.0166 |  | Fixed |  |
| Initial distribution of MI in Apixaban | 0.156 |  | Fixed |  |
| Initial distribution of stroke in Apixaban | 0.3552 |  | Fixed |  |
| Initial distribution of bleeding in Apixaban | 0.4209 |  | Fixed |  |

Abbreviations: ICH, intracranial hemorrhage; MI, myocardial infarction; SE, systemic embolism


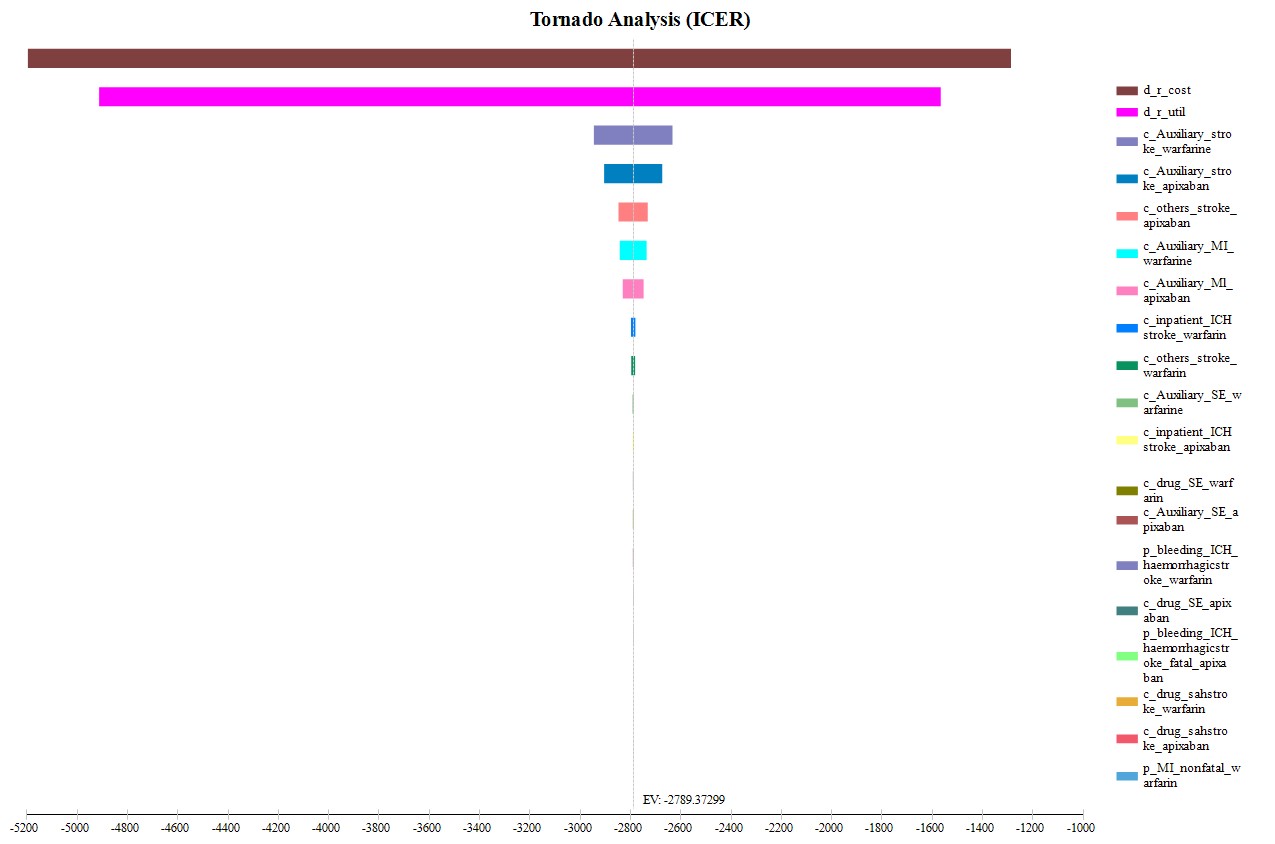


Supplementary Figure 1a. Results for one-way sensitivity analysis, the effect of parameter variation on the incremental cost (USD) per QALYs of Apixaban versus Warfarin (Tornado test)


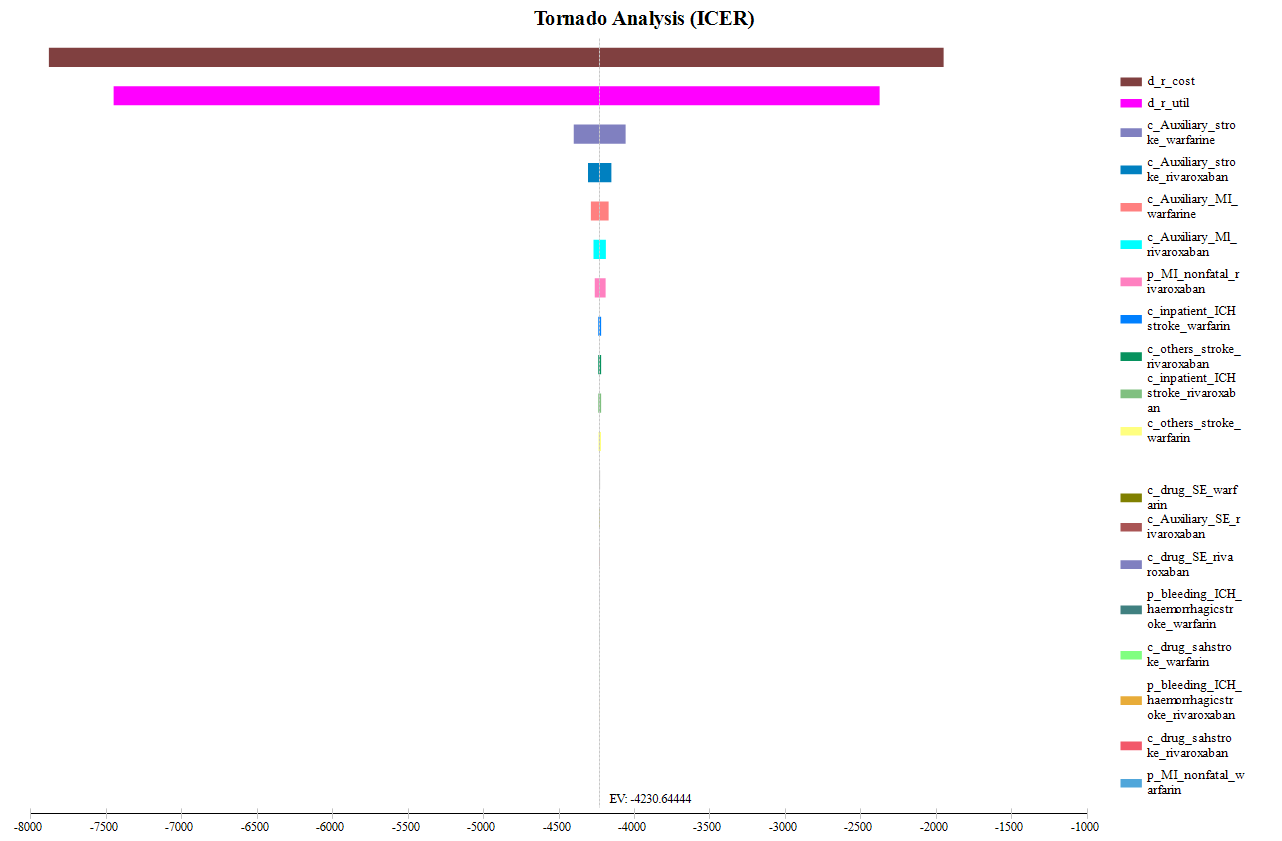


Supplementary Figure 1b. Results for one-way sensitivity analysis, the effect of parameter variation on the incremental cost (USD) per QALYs of Rivaroxaban versus Warfarin (Tornado test)

**
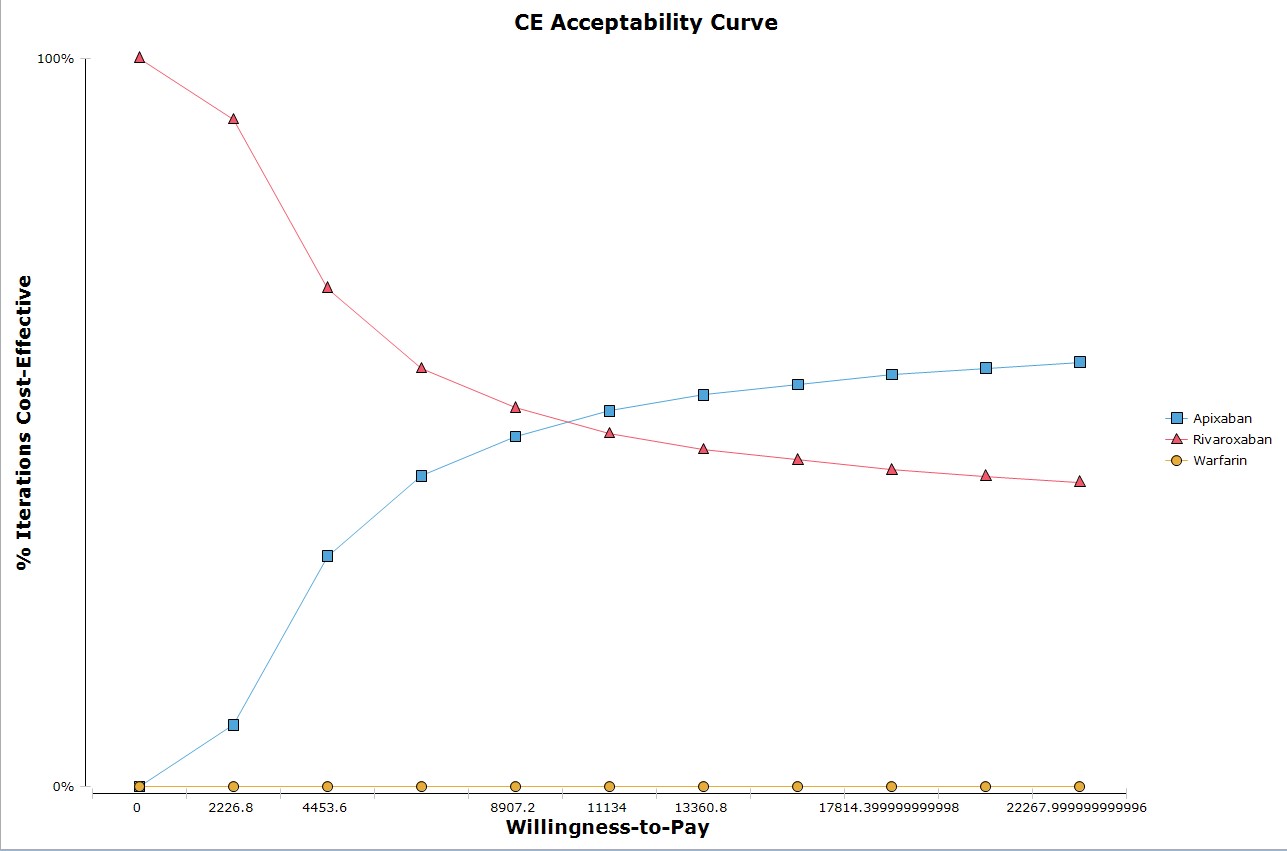
**

Supplementary Figure 2. Cost-effectiveness accessibility curve of Apixaban and Rivaroxaban vs. Warfarin for estimating the willingness to pay for Apixaban in patients with NVAF in Iran (Monte Carlo simulation)

Supplementary Figure 3a. Comparison of three different scenarios in budget impact analysis of Apixaban

Supplementary Figure 3b. Comparison of three different scenarios in budget impact analysis of Rivaroxaban
